# Autophagy Negatively Regulates Transmissible Gastroenteritis Virus Replication

**DOI:** 10.1038/srep23864

**Published:** 2016-03-31

**Authors:** Longjun Guo, Haidong Yu, Weihong Gu, Xiaolei Luo, Ren Li, Jian Zhang, Yunfei Xu, Lijun Yang, Nan Shen, Li Feng, Yue Wang

**Affiliations:** 1 State Key Laboratory of Veterinary Biotechnology, Harbin Veterinary Research Institute, Chinese Academy of Agricultural Sciences, Harbin, China; 2 Weike Biotechnology, Harbin Veterinary Research Institute, Chinese Academy of Agricultural Sciences, Harbin, China

## Abstract

Autophagy is an evolutionarily ancient pathway that has been shown to be important in the innate immune defense against several viruses. However, little is known about the regulatory role of autophagy in transmissible gastroenteritis virus (TGEV) replication. In this study, we found that TGEV infection increased the number of autophagosome-like double- and single-membrane vesicles in the cytoplasm of host cells, a phenomenon that is known to be related to autophagy. In addition, virus replication was required for the increased amount of the autophagosome marker protein LC3-II. Autophagic flux occurred in TGEV-infected cells, suggesting that TGEV infection triggered a complete autophagic response. When autophagy was pharmacologically inhibited by wortmannin or LY294002, TGEV replication increased. The increase in virus yield via autophagy inhibition was further confirmed by the use of siRNA duplexes, through which three proteins required for autophagy were depleted. Furthermore, TGEV replication was inhibited when autophagy was activated by rapamycin. The antiviral response of autophagy was confirmed by using siRNA to reduce the expression of gene p300, which otherwise inhibits autophagy. Together, the results indicate that TGEV infection activates autophagy and that autophagy then inhibits further TGEV replication.

Coronaviruses are enveloped, positive-stranded RNA viruses belonging to the family *Coronaviridae* in the order *Nidovirales*^1^. Coronaviruses are currently assigned to one of four genera on the basis of phylogenetic clustering: *alphacoronavirus, betacoronavirus, gammacoronavirus*, and *deltacoronavirus*^2^,^3^. The representative members in each genus are transmissible gastroenteritis virus (TGEV) from *alphacoronavirus*, mouse hepatitis virus (MHV) and severe acute respiratory syndrome virus (SARS-CoV) from *betacoronavirus*, infectious bronchitis virus (IBV) from *gammacoronavirus*, and Bulbut-CoV from *Deltacoronavirus*^4^. Members of this family cause acute and persistent infections and are broadly distributed among mammalian species (including humans) and avian species.

Coronaviruses infections are initiated by the binding of virons to cellular receptors. During infection, coronaviruses replicate in the host cytoplasm, where the viral genome becomes available for translation. The replicative structures of MHV form as double membrane vesicles (DMVs) in the cytoplasm^5^,^6^,^7^,^8^. DMVs are usually rare in normal cells, but large numbers of double-membrane structures called autophagosomes are usually induced by activation of the cellular autophagy machinery^9^. This suggests that DMVs induced by coronavirus infection are of autophagic origin. Autophagy is not only a cellular physiological pathway for self-degradation, i.e., a pathway for removal of damaged cellular components and for the degradation of unwanted products, but is also a defense mechanism that protects hosts against infection by bacteria^10^. Over the past several years, there has been a rapid increase in the number of studies, including the members of *Togviridae, Picornaviridae, Orthromyxoviridae* and *et al*., see reviews^11^,^12^,^13^,^14^,^15^,^16^,^17^.

The initial research on the coronavirus MHV showed that MHV infection induced autophagy, which is required for formation of DMVs-bound MHV replication complexes^18^. Further work using SARS-CoV and IBV also demonstrated the presence of the autophagosome protein marker LC3 or autophagy-like processes^19^,^20^. However, the work performed to date has focused mainly on the *betacoronaviruses* MHV and SARS-CoV and on the *gammacoronavirus* IBV. The involvement of the autophagy pathway in the replication of *alphacoronavirus*, like TGEV, remains to be investigated.

In this study, we have explored how TGEV infection affects autophagy and how autophagy affects TGEV infection. By monitoring autophagic signaling proteins and autophagic flux, we observed that TGEV infection induces autophagy. Furthermore, we examined the effect of the autophagy machinery on TGEV replication by modifying the autophagy pathway with pharmacological inducer/inhibitors and RNA interference. Of interest, we found that autophagy primarily functions to restrict TGEV replication.

## Results

### Activation of autophagic signaling following TGEV infection

**Figure 1:**
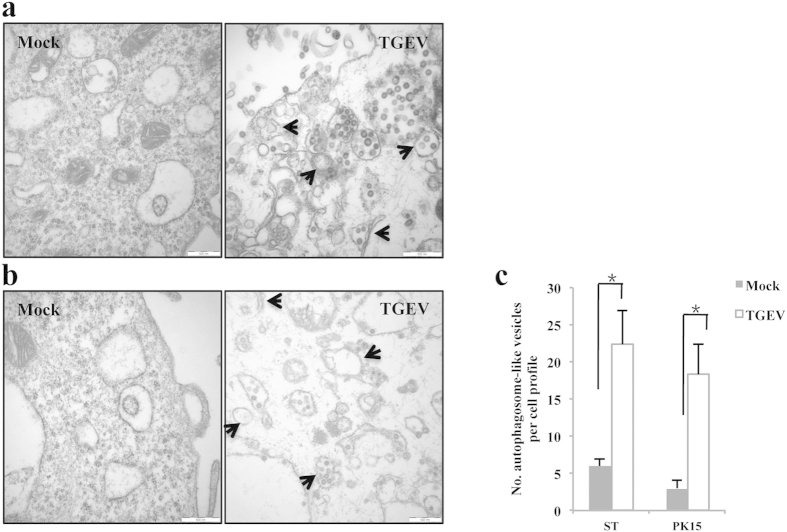
TGEV Infection leads to autophagic vesicle formation. ST cells (**a**) and PK15 cells (**b**) were mock-treated or infected with TGEV H165 at MOI of 1 for 24 h. Then the mock- and TGEV-treated cells were fixed and processed for electron microscopy analysis. The vesicles with the characteristics of autophagosome are indicated by black arrows in the relevant parts. Scale bars, 500 nm. (**c**) Quantification of the number of autophagosome-like vesicles per cell profile in mock- and TGEV-treated cells. Error bars, mean ± SD for 20 cells per experimental condition of three independent experiments. *p < 0.05. The *p* value is calculated using Student’s t-test.

Upon infection, the *betacoronavirus* MHV uses components of the cellular autophagic pathway to form DMVs in order to complete viral replication^18^. To determine whether TGEV infection regulates the cellular autophagy process by inducing the formation of DMVs, we used transmission electron microscopy (TEM) to examine the formation of autophagosome-like vesicles in TGEV-infected ST and PK15 cells. We observed that the number of double- and single-membrane vesicles increased near the perinuclear region of TGEV-infected ST cells, and that such structures were rare in the mock-treated samples (Fig. 1a). These autophagosome-like vesicles were morphologically identical to the MHV-infected Hela cells^6^. Similar results were obtained using PK15 cells (Fig. 1b). The number of autophagosome-like vesicles was greater in the cytoplasm of TGEV-infected cells than in the cytoplasm of mock-treated cells (Fig. 1c).

**Figure 2:**
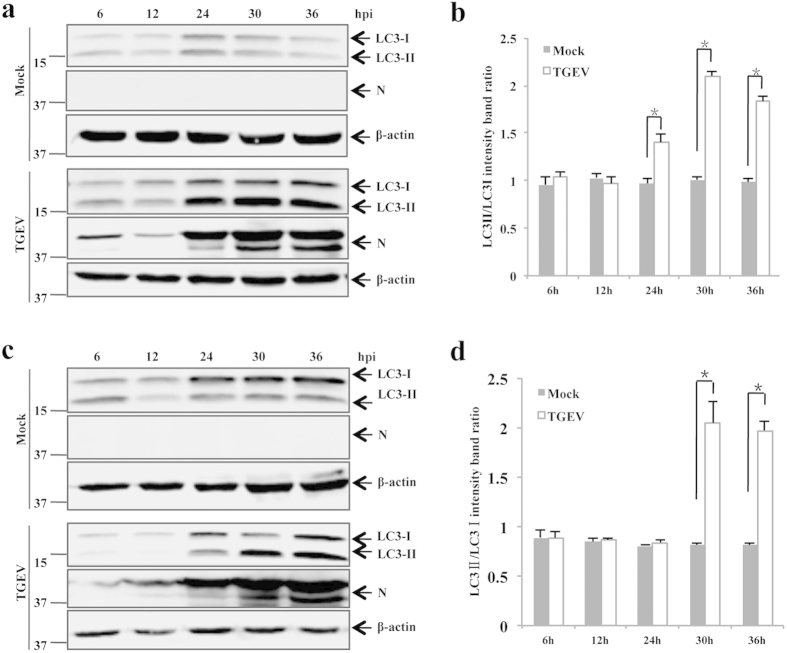
TGEV infection increases the conversion of LC3-I to LC3-II in ST and PK15 cells. ST cells (**a**) and PK15 cells (**c**) were mock-treated or infected with TGEV H165 at MOI of 1. At 6, 12, 24, 30, or 36 h post infection, cells were lysed, separated by reducing SDS-PAGE, and subjected to western blot with the antibodies against LC3, β-actin (loading control), or TGEV N protein as indicated. Densitometry was performed for quantification in ST cells (**b**) and PK15 cells (**d**). The ratio of LC3-II to LC3-I (LC3-II/LC3-I) from three independent experiments is expressed as mean ± SD. *p < 0.05. The *p* value is calculated using Student’s t-test. Gels were run under the same experimental conditions. For better clarity and conciseness of the presentation, cropped blots are shown. The raw uncropped images can be found in the Supplementary Fig. S1.

When autophagy is induced, a series of conjugation reactions lead to the conversion of cytosolic microtubule-associated LC3 (LC3-I) to the lipidated form LC3 (LC3-II), and the amount of LC3-II is correlated with the number of autophagosomes^21^,^22^. Therefore, we examined LC3 conversion by western blot assay using an anti-LC3 antibody that recognizes both forms of LC3 during TGEV infection. At the same time, mAb 3D7, which specifically recognizes TGEV nucleocapsid (N) protein, was used to estimate the infection progress. The results showed that the expression levels of LC3-II were upregulated in TGEV-infected ST cells relative to mock-treated cells (Fig. 2a). Because the ratio of LC3-II to LC3-I is regarded as an accurate indicator of autophagic activity^23^, we further assessed the densitometric ratios of LC3-II to LC3-I. As shown in Fig. 2b, the LC3-II to LC3-I ratio significantly increased from 24 to 30 h post infection (hpi) in TGEV-infected cells relative to controls; in addition, TGEV N protein was detected at 6 hpi and increased sharply between 24–30 hpi, which was consistent with the increase of LC3-II. By contrast, no obvious changes of the LC3-II to LC3-I ratio were observed in mock-treated cells (Fig. 2a,b). TGEV infection-induced autophagy was also assessed using PK15 cells. As was the case with ST cells, the relative amount of LC3-II significantly increased as TGEV infection of PK15 cells progressed over time, although the rate of increase in TGEV infection were slower in PK15 cells than in ST cells (Fig. 2c,d). Taken together, these data suggest that autophagosomes accumulated in TGEV-infected cells.

### Virus replication is required for the auotophagic process

**Figure 3:**
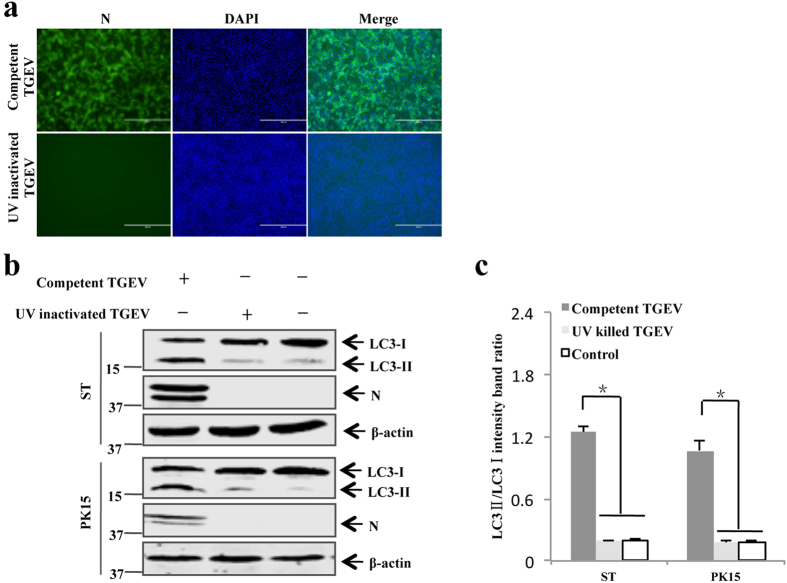
TGEV infection-induced autophagy is dependent of viral replication. (**a**) Immunofluorescence assay verified that UV-inactivated TGEV was replication defective. TGEV N protein was stained with mAb 3D7 and followed by FITC-conjugated goat anti-mouse IgG (green, left column). Nuclei counterstained with DAPI are visualized in blue (middle column). The right column illustrates a merged image of staining with mAb 3D7 and DAPI. (**b**) ST cells and PK15 cells were inoculated with replication competent TGEV or UV- inactivated TGEV at MOI of 10 for 24 h. Cells were lysed and analyzed by western blot with antibodies as indicated. (**c**) Densitometric data of LC3-II/LC3-I from three independent experiments are expressed as mean ± SD. *p < 0.05. The *p* value is calculated using Student’s t-test. Gels were run under the same experimental conditions. For better clarity and conciseness of the presentation, cropped blots are shown. The raw uncropped images can be found in the Supplementary Fig. S2.

The activation of autophagic activity by TGEV infection could be caused either by incoming virons or by viral replication products. To determine whether TGEV replication is required for the induction of autophagy, we challenged cells with either replication-competent or UV-inactivated virus and measured the effect on autophagy by monitoring the conversion of LC3-I to LC3-II. Before conducting the formal experiments, we used immunofluorescence assay (IFA) to verify that the UV-inactivated virus was replication defective (Fig. 3a). By using western blot, we found that the levels of both LC3-I and LC3-II were similar in ST cells inoculated with UV-inactivated TGEV at MOI of 10 and in mock-treated ST cells at 24 h post inoculation; in addition, no N protein synthesis was detected in ST cells inoculated with UV-inactivated TGEV. In contrast, ST cells infected with replication-competent TGEV apparently underwent the conversion of LC3-I to LC3-II (Fig. 3b,c). Similar results were obtained with PK15 cells (Fig. 3b,c), suggesting that TGEV replication is required for the formation of autophagosomes.

### TGEV infection enhances autophagic flux

**Figure 4:**
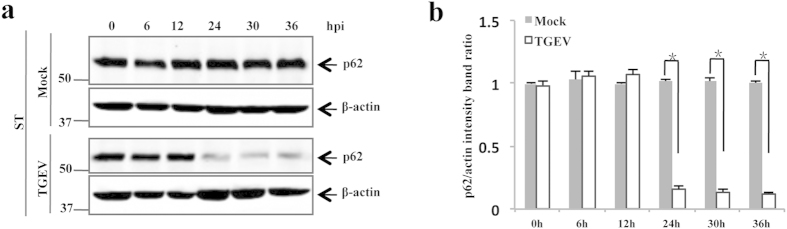
TGEV infection enhances the autophagic flux. (**a**) ST cells were mock-treated or TGEV-infected at MOI of 1. At the indicated time points post infection, mock- and TGEV-infected cells were lysed and blotted with antibody against p62 or β-actin as indicated. (**b**) Densitometric data of p62/β-actin in ST cells from three independent experiments are expressed as mean ± SD. *p < 0.05. The *p* value is calculated using Student’s t-test. Gels were run under the same experimental conditions. For better clarity and conciseness of the presentation, cropped blots are shown. The raw uncropped images can be found in the Supplementary Fig. S3.

To determine whether autophagosome accumulation is due to autophagy induction or to a block in the maturation of autophagosome into autolysosomes^24^,^25^, we performed autophagic flux assays, which can distinguish between these two possibilities^26^. Because p62/SQSTM1 serves as a link between LC3 and ubiquitinated substrates, and because inhibition of autophagy leads to an increase in the levels of p62, p62 degradation is considered an indicator of autophagic flux^22^,^27^. Therefore, we used a western blot assay to measure the changes in p62 protein levels upon virus infection. As shown in Fig. 4a,b, TGEV infection increased degradation of p62 in ST cells over time. There was no significant alteration of p62 between 0 h and 36 h in the mock-treated control. These data indicate that the accumulation of autophagosomes induced by TGEV infection is due to autophagic activation rather than to a block in autophagosome maturation.

**Figure 5:**
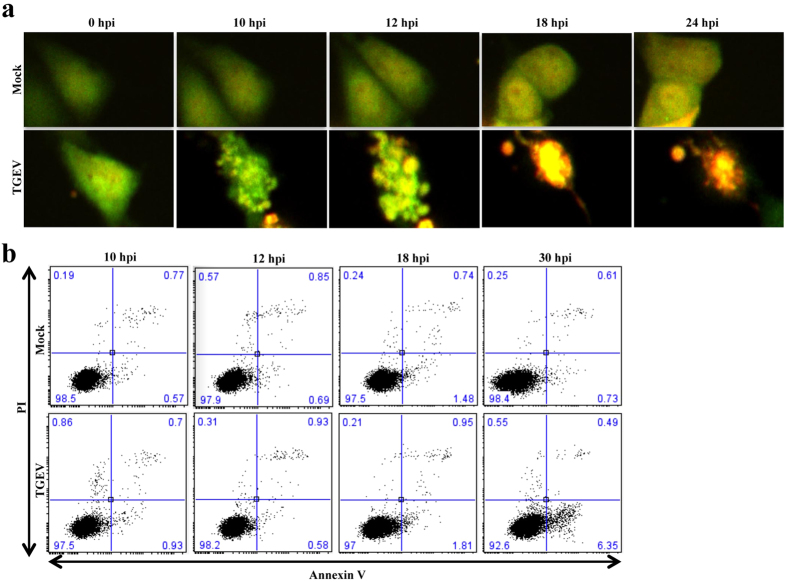
TGEV infection facilitates autophagosome maturation. (**a**) ST cells were transfected with plasmid GFP-RFP-LC3 for 24 h and followed by infection with mock or TGEV. Cells were cultured under the live cell imaging system and the dynamic behavior of autophagy is captured in real time in live cells. Imaging was performed at 1 frame per 10 min, and a selected interval within this sequence is shown, starting right after virus infection. (**b**) Effects of TGEV infection on apoptosis of ST cells. Cells were harvested post TGEV infection at indicated time points and analyzed with FITC Annexin V Apoptosis Detection Kit I according to the manufacturer’s instructions. Percentage of TGEV-induced ST apoptotic cells was listed as indicated. Data are representative of at least three independent experiments.

To further monitor the autophagic flux upon viral infection, we evaluated the autophagosome maturation process by transfecting cells with a tandem reporter construct GFP-RFP-tagged LC3 (GFP-RFP-LC3). The fusion protein GFP-LC3 is relatively stable, but the low pH in the autolysosome quenches the GFP fluorescent signal. RFP-LC3, in contrast, exhibits more stable fluorescence in acidic compartments, which makes the GFP-RFP-LC3 tandem construct useful for assessing autophagosome dynamics^28^,^29^. Therefore, ST cells were transfected with GFP-RFP-LC3 plasmid and then were infected with TGEV before live cell imaging was used to examine the dynamics of autophagosome maturation. In TGEV-infected cells, the GFP-LC3 and RFP-LC3 puncta formed a yellow autophagosome by the 10-h frame (Fig. 5a). Subsequently, the yellow puncta turned red, most likely as a result of the quenching of GFP-LC3 fluorescence in the acidic amphisomes or autolysosomes (Fig. 5a & movie, data not shown); this change in color indicated the maturation of autophagosomes. In mock-treated cells, in contrast, the few yellow or red LC3 puncta represent the baseline range of autophagic flux. To confirm that the autophagic puncta are not derived from apoptotic blebs, we performed apoptosis staining during the infection. As shown in Fig. 5b, there was no increase in the percentage of apoptotic cells at 10, 12, and 18 hpi in infected cells relative to mock-treated cells; at 30 hpi, some virus-infected cells began to be apoptosis positive. Collectively, the results demonstrate that TGEV infection leads to the degradation of p62 protein via the autophagic-lysosomal pathway, suggesting that virus infection significantly increases autophagic flux.

### Pharmacological inhibition of autophagy increases the replication of TGEV

**Figure 6:**
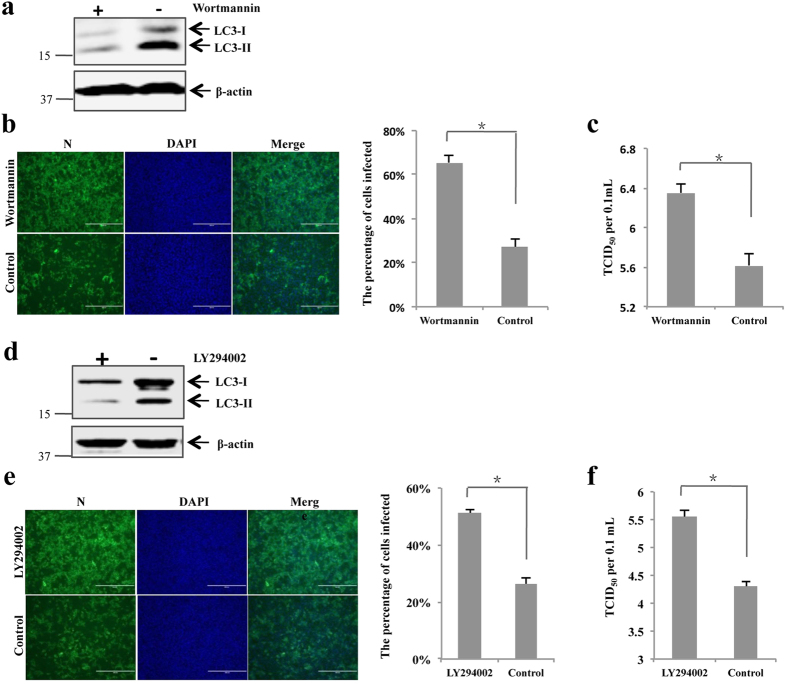
Pharmacological inhibition of autophagy enhances TGEV replication. (**a**,**d**) ST cells were treated with wortmannin (3 μM), LY294002 (25 μM) or DMSO carrier control for 4 h prior to TGEV infection. At 24 h post infection, cells were lysed and subjected to western blot with the antibodies against LC3 or β-actin. (**b**,**e**) Immunofluorescence assay to detect TGEV with mAb 3D7. Cells were treated with control or autophagy inhibitors for 4 h indicated, followed by infection with TGEV H165 at MOI of 1. At 24 h after infection, cell monolayer was stained for TGEV N protein expression and nuclei counterstained. The percentage of TGEV-infected cells per view from three independent experiments is expressed as mean ± SD. (**c**,**f**) TGEV titers on ST cells treated with wortmannin, LY294002, or control. The progeny virus titers were calculated and expressed as TCID_50_ per 0.1 ml. Results represent the mean ± SD for three independent experiments. *p < 0.05. The *p* value is calculated using Student’s t-test. Gels were run under the same experimental conditions. For better clarity and conciseness of the presentation, cropped blots are shown. The raw uncropped images can be found in the Supplementary Fig. S4.

Given that autophagy is induced by TGEV infection, we then determined whether cellular autophagy regulates TGEV replication. To accomplish this, we exposed the cells to wortmannin or LY294002, both of which can inhibit autophagy at an early stage by inhibiting the phosphatidylinositol 3-kinase (PI3K) pathway^30^. As shown in Fig. 6a,d, wortmannin and LY294002 pretreatment reduced the conversion of LC3-I to LC3-II in TGEV-infected ST cells, indicating that the virus-induced autophagy is inhibited by these two compounds. As indicated by IFA, treatment of cells with either wortmannin (Fig. 6b) or LY294002 (Fig. 6e) increased the percentage of infected cells by 3- or 2-fold, respectively, relative to untreated cells. An increase in the titers of progeny virus in the cells treated with wortmannin or LY294002 was confirmed by measuring the median tissue culture infectious dose (TCID_50_) (Fig. 6c,f). These data indicate that virus-induced autophagy may protect cells from TGEV infection.

### Depletion of endogenous LC3, ATG5, and ATG7 enhances viral replication

**Figure 7:**
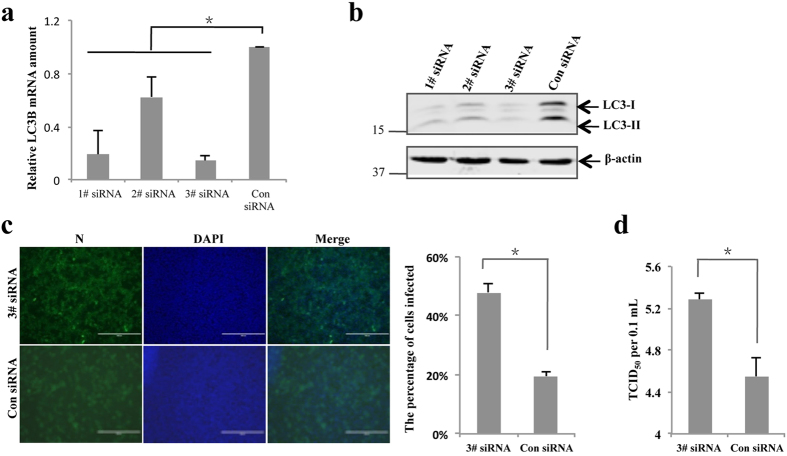
Inhibition of autophagy with specific siRNA targeting endogenous LC3 enhances TGEV replication. (**a**) ST cells were transfected with siRNA duplexes against porcine LC3 or nontargeting control siRNA, as described in Material and Methods. Knockdown efficiency of LC3 siRNA duplexes was indicated by relative LC3 mRNA to β-actin mRNA when siRNA duplexes at the concentration of 100 nM. (**b**) Detergent lysate from equivalent numbers of ST cells treated with LC3-specific or nontargeting siRNA were subjected to reducing SDS-PAGE and immunoblotting with antibodies to LC3 or β-actin (loading control). (**c**) ST cells were transfected with LC3-specific siRNA duplexes 3# or control siRNA at the concentration of 100 nM for 24 h followed by infection with TGEV. At 24 h post infection, cell monolayer was fixed and examined the TGEV infection by IFA as described in Fig. 6b legend. The percentage of TGEV-infected cells per view from three independent experiments is expressed as mean ± SD. (**d**) Determination of TGEV replication in LC3-specific siRNA-treated cells. The extracellular virus yields were determined at 24 hpi and expressed as TCID_50_ per 0.1 ml. Representative data from three independent experiments are shown as the mean ± SD. *p < 0.05. The *p* value is calculated using Student’s t-test. Gels were run under the same experimental conditions. For better clarity and conciseness of the presentation, cropped blots are shown. The raw uncropped images can be found in the Supplementary Fig. S5.

**Figure 8:**
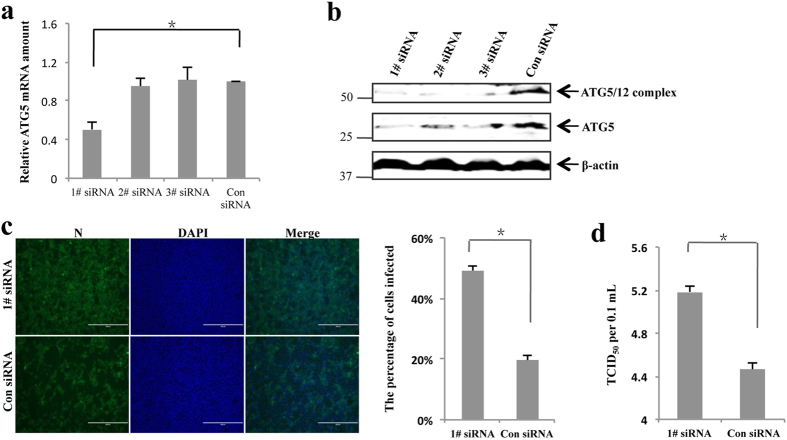
Inhibition of autophagy with specific siRNA targeting endogenous ATG5 enhances TGEV replication. (**a**) ST cells were transfected with siRNA duplexes against porcine endogenous ATG5 or nontargeting control siRNA for 24 h. Knockdown efficiency of specific siRNA was indicated by relative ATG5 mRNA to β-actin mRNA when siRNA duplexes at the concentration of 100 nM. (**b**) Detergent lysate from equivalent numbers of siRNA transfected-ST cells were subjected to immunoblotting with antibodies as indicated. (**c**) ST cells were transfected with either ATG5-specific siRNA duplexes 1# or control siRNA. At 24 h after transfection, cells were infected with TGEV at MOI of 1. Cells were fixed at 24 hpi and examined the TGEV infection by IFA as described in Fig. 6b. The percentage of TGEV-infected cells per view from three independent experiments is expressed as mean ± SD. (**d**) The extracellular virus yields were determined at 24 hpi and expressed as TCID_50_ per 0.1 ml. Representative data from three separate experiments are shown as the mean ± SD of three separate experiments. *p < 0.05. The *p* value is calculated using Student’s t-test. Gels were run under the same experimental conditions. For better clarity and conciseness of the presentation, cropped blots are shown. The raw uncropped images can be found in the Supplementary Fig. S6.

**Figure 9:**
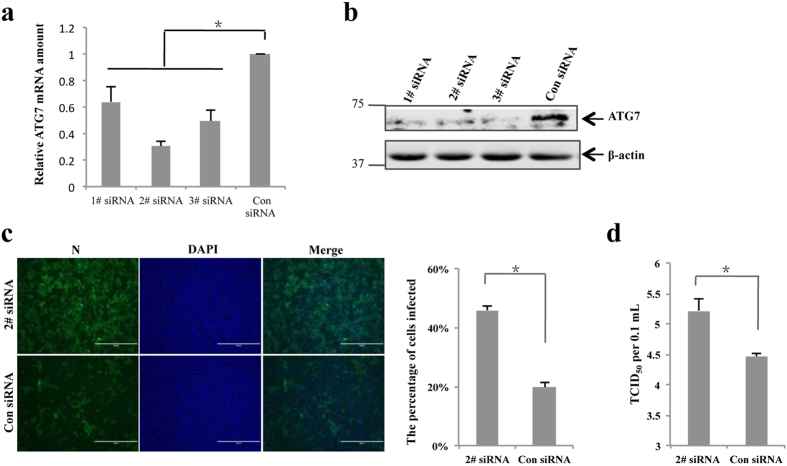
Inhibition of autophagy with specific siRNA targeting endogenous ATG7 enhances TGEV replication. (**a**) ST cells were transfected with siRNA targeting porcine endogenous ATG7 or control siRNA for 24 h, and followed by mock treatment or TGEV infection at MOI of 1. At 24 hpi, the silencing efficiency of ATG7 siRNA was analyzed. (**b**) Detergent lysate from equivalent numbers of ST cells transfected with ATG7-specific siRNA were subjected to immunoblotting with antibodies to ATG7 or β-actin. (**c**) ST cells were transfected with ATG7-specific siRNA duplexes 2# or control siRNA for 24 h followed by infection with TGEV. At 24 h post infection, TGEV infection was examined by IFA. The percentage of TGEV-infected cells per view from three independent experiments is expressed as mean ± SD. (**d**) Virus titers in ATG7-siRNA 2#-transfected ST cells were determined by TCID_50_ assay. Representative data from three independent experiments are shown as the mean ± SD. *p < 0.05. The *p* value is calculated using Student’s t-test. Gels were run under the same experimental conditions. For better clarity and conciseness of the presentation, cropped blots are shown. The raw uncropped images can be found in the Supplementary Fig. S7.

In additional experiments with pharmalogical regulators, we used siRNA duplexes that target three highly conserved genes required for mammalian autophagy (LC3, ATG5, and ATG7)^31^,^32^,^33^. These three genes were examined to confirm that the effects observed were due to the autophagy pathway rather than to the non-target effects of the siRNA. To deplete the proteins, we treated ST cells with siRNA duplexes for 24 h and then challenged the cells with TGEV. When cells were separately treated with each of the three siRNA duplexes, the levels of LC3, ATG5, and ATG7 mRNA were decreased in each case relative to levels in cells treated with nontargeting control siRNA (Figs 7a, 8a and 9a). Western blot analysis of detergent lysates collected from cells revealed that levels of endogenous LC3, ATG5, or ATG7 protein were significantly lower in cells treated with the siRNA duplexes than in cells treated with nontargeting control siRNA (Figs 7b, 8b and 9b). Because the data indicated that knockdown efficiency was greatest with LC3-specific siRNA duplexes 3#, ATG5-specific siRNA duplexes 1#, and ATG7-specific siRNA duplexes 2#, these specific siRNA duplexes were used in the following experiments. Following the knockdown of each autophagy-related gene with these gene-specific siRNA duplexes, we observed an increase in the percentage of TGEV-infected cells in comparison to the nontargeting control siRNA (Figs 7c, 8c and 9c). We also found that autophagic protein depletion led to increased viral titers (Figs 7d, 8d and 9d). Together, the data demonstrate that inhibition of autophagy promotes TGEV replication.

### Induction of autophagy by rapamycin reduces viral replication

**Figure 10:**
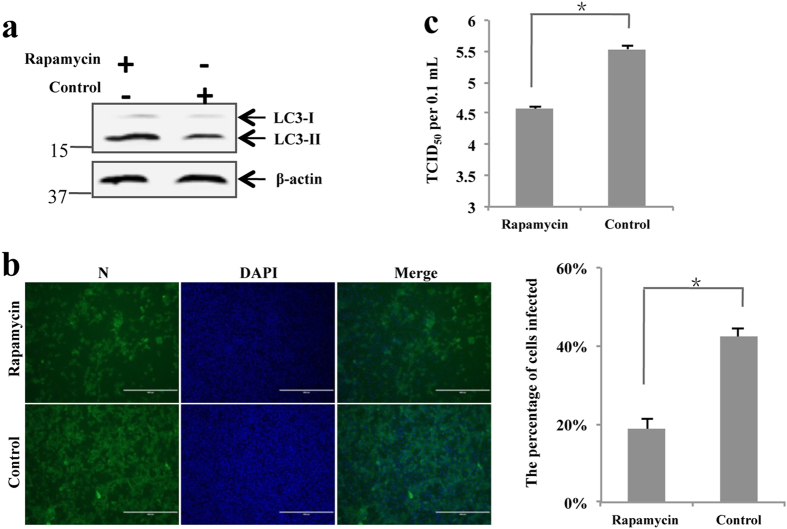
Induction of autophagy with rapamycin restricts TGEV replication. (**a**) Induction of autophagy with rapamycin in ST cells. ST cells were treated with rapamycin at the concentration of 100 nM for 4 h prior to TGEV infection. Cells were lysed and analyzed by western blot with antibodies against LC3 or β-actin. (**b**) At 24 hpi, cells were fixed and examined the TGEV infection by IFA. The percentage of TGEV-infected cells per view from three independent experiments is expressed as mean ± SD. (**c**) Virus titer was determined in rapamycin-treated ST cell. Representative data from three independent experiments are shown as the mean ± SD. *p < 0.05. The *p* value is calculated using Student’s t-test. Gels were run under the same experimental conditions. For better clarity and conciseness of the presentation, cropped blots are shown. The raw uncropped images can be found in the Supplementary Fig. S8.

To confirm that autophagy inhibits TGEV infection, we treated ST cells with rapamycin, which induces autophagy by blocking the mammalian target of the rapamycin pathway^34^,^35^. As shown in Fig. 10a, rapamycin treatment increased the amounts of LC3-II in TGEV-infected ST cells, indicating that autophagy is upregulated by rapamycin in virus-infected cells. Using IFA, we observed that the percentage of TGEV-infected cells was lower in the rapamycin-treated cells than in nontreated cells (Fig. 10b). Viral yields were also lower in rapamycin-treated cells than in nontreated cells (Fig. 10c). These observations suggest that autophagy restricts TGEV replication and reduces viral yields, which is consistent with the results of our autophagy inhibition assays.

### Knockdown of the endogenous acetyltransferase p300 protein reduces TGEV replication

**Figure 11:**
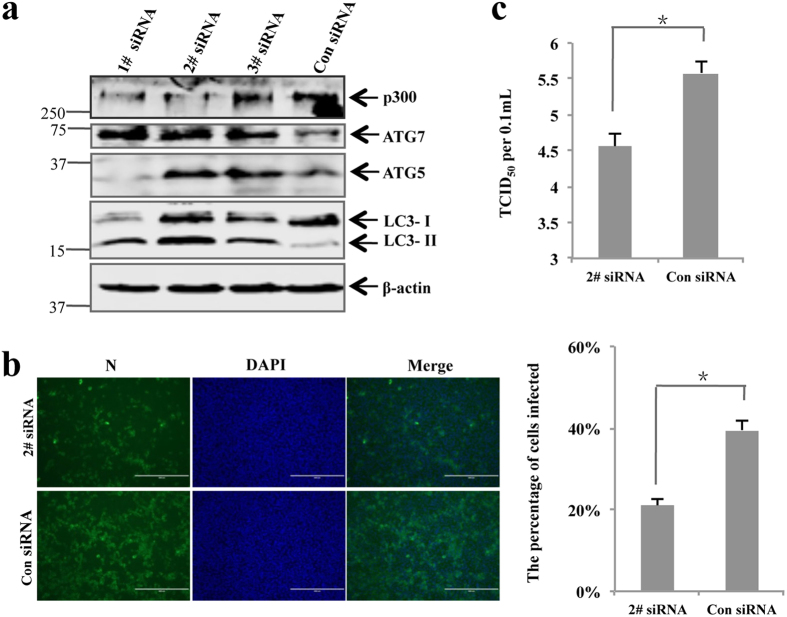
Induction of autophagy with specific siRNA targeting endogenous acetyltransferase p300 reduces TGEV replication. (**a**) Autophagy is inhibited with specific siRNA targeting p300. ST cells were transfected with siRNA duplexes against porcine endogenous p300 or control siRNA at the concentration of 100 nM. Detergent lysate from equivalent numbers of siRNA duplexes transfected ST cells were subjected to reducing SDS-PAGE and immunoblotting with antibodies to p300, LC3, ATG5, ATG7 or β-actin. (**b**) ST cells were transfected with either specific siRNA duplexes 2# or control siRNA at the concentration of 100 nM for 24 h followed by infection with TGEV. At 24 hpi, cells were fixed and examined the TGEV infection by IFA. The percentage of TGEV-infected cells per view from three independent experiments is expressed as mean ± SD. (**c**) At 24 hpi, the extracellular virus titers in p300 siRNA 2#-transfected cells were measured by TCID_50_. Representative data from three independent experiments are shown as the mean ± SD. *p < 0.05. The *p* value is calculated using Student’s t-test. Gels were run under the same experimental conditions. For better clarity and conciseness of the presentation, cropped blots are shown. The raw uncropped images can be found in the Supplementary Fig. S9.

To confirm that the results we obtained with rapamycin reflected autophagy induction rather than other processes, we determined whether depletion of endogenous acetyltransferase p300 by siRNA also affects TGEV infection. Previous research demonstrated that acetyltransferase p300 can degrade LC3, ATG5, and ATG7 by post-translational modifications in Hela cells, and that knockdown of p300 can accelerate autophagy^36^. We treated ST cells with p300 siRNA duplexes for 24 h and then infected them with TGEV. As shown in Fig. 11a, p300 siRNA-transfected ST cells had decreased levels of the endogenous acetyltransferase p300 protein and increased levels of LC3-II, ATG5, and ATG7 proteins, suggesting that p300 negatively regulates autophagy in ST cells. In addition, knockdown efficiency was greater for p300 siRNA duplexes 2# than for the other duplexes, and p300 siRNA duplexes 2# was therefore used for further study. Using IFA, we found that the percentage of TGEV-positive cells was lower in cells treated with the p300 siRNA duplexes 2# than in cells treated with nontargeting control siRNA (Fig. 11b). The reduction in viral replication determined by IFA was verified by measuring the TCID_50_ (Fig. 11c). Overall, these results demonstrate that autophagy plays an antiviral role during TGEV infection.

## Discussion

By contributing to self-degradation, autophagy helps maintain cellular homeostasis and increases cell survival. Studies of cellular autophagy have then increased greatly over the past two decades^37^. Subsequently, studies about relationship between autophagy and viral infection have rapidly emerged^38^. To date, more than 50 virus species have been investigated, and the results indicate that autophagy inhibits the replication of some viruses but promotes the replication of others^39^,^40^. However, the relationship between TGEV infection and autophagy had not been investigated before the current research. In the present work, we investigated the interaction between the cellular autophagy pathway and TGEV infection. We report for the first time that TGEV infection induces autophagy and that autophagy limits TGEV replication.

TGEV, which is the representative member of *alphacoronavirus*, infects the enteric and respiratory tissues of newborn piglets, resulting in nearly 100% mortality^41^,^42^. Although this virus was identified 30 years ago^43^, the interactions between TGEV replication and host cell responses are incompletely understood. Previous studies revealed that infection by several other coronaviruses, including MHV, SARS-CoV, IBV, and Middle East respiratory syndrome coronavirus, induced the formation of DMVs^6^,^18^,^44^,^45^,^46^,^47^. In the present study, TEM revealed that the formation of DMVs, which is a hallmark of coronavirus replication, was greatly induced in both ST and PK15 cells during TGEV infection. The presence of DMVs suggested that this virus might induce the accumulation of autophagic vesicles in TGEV-infected cells. Using immunoblotting to evaluate LC3 modification, we then confirmed that the induction of DMVs by TGEV infection was related to the autophagic process. Additionally, we found that viral replication is required in TGEV-induced autophagy. Although DMVs exist in the TGEV-infected cells, we did not know whether this *alphacoronavirus* species, like *betacoronavirus* species, hijacks the autophagosome machinery to facilitate virus replication^46^. Further work is needed to conclusively answer this question. Nevertheless, our findings clearly demonstrate that TGEV infection induces the accumulation of autophagosomes in target cells, and that the TGEV replication process is required for the induction of autophagic activity.

The cellular autophagic process has been investigated for several viruses^48^. Previous reports showed that the induction of autophagy by viruses can be due to the formation of autophagosomes or to the inhibition of phagosome maturation^24^,^25^. Another way to verify the cellular state of autophagy is to monitor autophagic flux, which can be measured by detecting the levels of p62 and the transportation of LC3^22^,^27^. Our immunoblotting results showed that TGEV infection decreases the expression level of p62 protein, indicating that TGEV infection enhances the autophagic flux in target cells. Moreover, by using a GFP-RFP-LC3 tandem construct to morphologically trace autophagic flux, we assessed the maturation of autophagosomes into autolysomes; the results indicated that the accumulation of autophagosomes is due to their *de novo* formation and not to the inhibition of their maturation. These data demonstrate that there is a potential relationship between the autophagic process and TGEV infection.

The autophagy pathway has been reported to be a critical component in development, survival, and the immune response^49^. The antiviral potential of autophagy has recently been studied^14^,^16^,^50^. The first-identified mammalian autophagy protein, Beclin 1, protects the host against infection by Sindbis virus (a positive-stranded RNA virus) by reducing viral titres^51^. A similar antiviral role for autophagy has been described in response to infection by α-herpesvirus-1, which is a double-stranded DNA virus^52^,^53^. An antiviral function of autophagy has also been found in another RNA virus, vesicular stomatitis virus (VSV)^54^. In the present study, we pharmacologically inhibited the formation of autophagosomes and consequently increased virus replication; this finding was confirmed by siRNA knockdown of three highly conserved endogenous genes required for autophagy. These results are consistent with the role of autophagy in cell-autonomous defense against microbial invasion^8^,^55^,^56^. Accordingly, we found that inhibition of autophagy increases TGEV replication.

Recent studies show that several members of the *Coronaviridae* family, including IBV, MHV, and SARS-CoV, can induce autophagy but do not require autophagy for replication in host cells^46^,^57^,^58^. However, Prentice *et al*. reported that MHV utilizes the cell’s autophagic machinery to facilitate virus replication^18^. These observations suggest that replication mechanisms may differ among viruses of the same family and even of the same species; these differences may be related to differences in cell-type specificity or in mechanisms of infection. Therefore, to determine whether the induction of autophagy is an anti-TGEV response, we examined the effect of controlled autophagosomal activation on virus replication. Our data showed that rapamycin treatment induced autophagy, which then inhibited virus replication, suggesting that pharmacological modulation of autophagy might be a useful way to restrict TGEV replication.

To further evaluate the effect of autophagy modulation on virus replication, we attempted to genetically adjust autophagy. Lee and Finkel found that acetyltransferase p300 is involved in the acetylation of several known components of the autophagy machinery, including ATG5, ATG7, ATG8 (LC3), and ATG12; acetyltransferase p300 degrades these autophagy-dependent proteins and inhibits autophagosome formation under starved conditions^36^. In addition, Huang *et al*. reported that TGEV infection downregulates acetyltransferase p300 expression and increases cell apoptosis^59^, suggesting that acetyltransferase p300 is regulated upon TGEV infection. When we knocked down the endogenous p300 in ST cells with specific siRNA duplexes, autophagy was induced, which is consistent with previous findings^36^, and TGEV replication was reduced. These data indicate that induction of autophagy restricts viral replication. Based on these results, we conclude that autophagy has an antiviral rather than a proviral role in TGEV infection, although the detailed interplay between the autophagic machinery and TGEV and other viruses remains incompletely understood.

In summary, autophagy has been shown to be essential for defense against a variety of pathogens in cell culture. Here, we demonstrate for the first time that TGEV infection triggers the formation of autophagosomes. Down-regulation of the autophagy signal pathway increases viral replication, and up-regulation of the autophagic process reduces viral replication. In addition, the involvement of acetyltransferase p300 in the regulation of TGEV replication is reported for the first time. The antiviral pathway identified in this study may play a role in the control of other infectious agents. Finally, pharmacological modulation of the autophagic pathway should be explored as a means to inhibit viral replication.

## Material and Methods

### Cells and virus

ST and PK15 cells from ATCC were cultured in Dulbecco’s modified Eagle’s medium (DMEM; Life Technologies, 11995) supplemented with 10% fetal bovine serum (FBS, Hyclone Laboratories Inc.). TGEV strain H165 was propagated in ST cells cultured in DMEM medium supplemented with 5% FBS^60^. To obtain replication-incompetent TGEV, 10 ml of virus suspension was dispensed to form a layer of fluid in an open, cell culture dish and was irradiated with UV light for 5 h by gentle shaking at intervals. The absence of virus infectivity after UV treatment was confirmed by immunofluorescence assay.

### Antibodies and reagents

Rapamycin (R0395), wortmannin (W1628) and LY294002 (L9908), rabbit anti-LC3 (L7543), and rabbit anti-p62/SQSTM1 (P0067) polyclonal antibodies were purchased from Sigma-Aldrich. Fluorescein isothiocyanate (FITC)-conjugated goat anti-mouse IgG (115-096-146) was purchased from Jackson ImmunoResearch. Goat anti-ATG5 polyclonal antibody (N-18 sc-8666), goat anti-ATG7 polyclonal antibody (N-20, sc-8668), rabbit anti-p300 (N-15, sc-585) and anti-β-actin polyclonal antibodies (sc-47778) were obtained from Santa Cruz Biotechnology. IRDye 800CW donkey anti-goat IgG (926-32214), IRDye 800CW goat anti-rabbit IgG (926-32211) and IRDye 680RD goat anti-mouse IgG (926-68070) were purchased from Li-Cor. The mAb 3D7 against TGEV N protein was prepared in our laboratory. siRNA duplexes from Sigma-Aldrich, specifically to knock down the associated gene expression including LC3, ATG5, ATG7, p300 and unrelated gene, were listed in Table 1.

### Viral infection and drug treatment

ST and PK15 cells were infected with TGEV at MOI of 1 for 1 h at 37 °C. Unattached viruses were removed and the cells were washed three times with phosphate buffered saline (PBS). The cells were then cultured in complete medium for various time points until samples had been harvested. For the autophagy induction and inhibition experiments, ST cells were pretreated with wortmannin (3 μM), LY294002 (25 μM), rapamycin (100 nM), or equal volume of DMSO (carrier control) for 4 h prior to viral infection. Virus incubation was carried out at 37 °C for 1 h. After three times washing with PBS, the cells were reinoculated with fresh media containing wortmannin, LY294002, rapamycin, or DMSO during treatments. At 24 h post infection, cells were collected for subsequent analysis.

### Transmission electron microscopy

For our conventional ultrastructural analysis, ST and PK15 cells were either mock-treated or infected with TGEV at MOI of 1 for 24 h. The cells were then fixed and processed for section preparation as described previously^61^. Images of the ultrathin sections were viewed on an H-7650 transmission electron microscope (Hitachi, Japan). Autophagosome-like vesicles were defined as double- or single-membrane vesicles measuring 0.3 to 2.0 μm in diameter with clearly recognizable cytoplasmic contents. The number of autophagosome-like vesicles was calculated in ST and PK15 cells post infection.

### SDS-PAGE and western blot

Cells were detergent-lysed and performed as previously described^62^. Typically, samples were separated by SDS-PAGE under reducing conditions and transferred onto a PVDF membrane. Membranes were blocked with 2% bovine serum albumin (BSA; Sigma, A7906) in PBS for 60 min and then incubated with a primary antibody for 60 min. After three times washing with PBS + 0.05% Tween-20, the membranes were incubated with IRDye-conjugated secondary antibody (Li-Cor Biosciences, Lincoln, NE) diluted in washing buffer for 60 min. Membranes were washed as described above, and subjected to scanning on an Odyssey instrument (Odyssey infrared imaging system, Li-Cor Biosciences).

### Immunofluorescence assay (IFA)

IFA was performed as previously described^63^. Cells were subjected to infection with TGEV after treatments as indicated, followed by fixation in 33.3% acetone for 30 min at room temperature and drying. Fixed cells were incubated with mouse anti-TGEV N protein mAb 3D7 and then incubated with FITC-conjugated goat anti-mouse IgG. Finally, the cells were visualized under an Olympus inverted fluorescence microscope equipped with a camera following by counterstaining with 5 μg/ml 4′,6-diamidino-2-phenylindole (DAPI). The percentage of virus-positive cells was calculated.

### Quantitative RT-PCR

At 24 h post transfection of siRNA duplexes, total RNA was extracted from cells using RNeasy RNA purification kit (Qiagen) to generate cDNA with oligo (dT) primers using Superscript® II reverse transcriptase (Invitrogen, USA). Quantitative RT-PCR reactions were set up in triplicate using SYBR premix Ex Taq (TaKaRa) and 10 μM specific primers (Table 2), with the following cycling program: 15 s at 94 °C, 40 cycles of 5 s at 94 °C, 20 s at 55 °C and 25 s at 72 °C. Relative quantification was performed by the cycle threshold (∆∆CT) method^63^. Briefly, CT values were normalized to β-actin mRNA (β-actin, internal standard), as ∆CT, which was determined by the formula ∆CT = CT (target gene) −CT (β-actin). Fold changes was determined by 2^−∆∆CT^, where ∆∆CT = ∆CT (siRNA1-3#) −∆CT (Control siRNA).

### Live cell imaging

ST cells were transfected with the GFP-RFP-LC3 plasmid for 24 h prior to TGEV infection at MOI of 1. Subsequently the cells were recorded under the Ultra View Vox 3D live cell imaging system (PerkinElmer). By using this confocal imaging, three-dimensional data can be captured by collecting two-dimensional images in the XY plane at a range of Z-plane settings. Due to low levels of light the three-dimensional data can be captured in real time without damaging the cells, so that autophagic processes can be recorded as they happen. When the images have been captured, we can use the Volocity analysis tool (Ultra View Vox, PerkinElmer) to extract data and quantify cellular processes. Images and data can then be transferred to video program for display.

### Apoptosis analysis

ST cells were treated with mock or TGEV at MOI of 1. At indicated time points cells were stained with FITC Annexin V Apoptosis Detection Kit I (BD Biosciences, USA) and analyzed by flow cytometry as described in the manufacturer’s instructions.

### Statistical analysis

Variables are expressed as mean ± S.D. Statistical analyses were performed using student’s t test and analyzed using Excel. A *p* value of < 0.05 was considered significant.

## Additional Information

**How to cite this article**: Guo, L. *et al*. Autophagy Negatively Regulates Transmissible Gastroenteritis Virus Replication. *Sci. Rep.* **6**, 23864; doi: 10.1038/srep23864 (2016).

## Supplementary Material

Supplementary Information

## Figures and Tables

**Table 1:** Sequences of sense strands of double-stranded RNA used to ablate specific protein expression in ST cells.

Target	siRNA	Sequence (5′→3′)
LC3	1# siRNA	GAGUGAGCUCAUCAAGAUA
	2# siRNA	CUCAGGAGACUUUCGGAAU
	3# siRNA	CGAUUUGUGAGGUGUACGA
ATG5	1# siRNA	GCUCUUCCUUGGAACAUCA
	2# siRNA	CUGAUGCUUUAAAGCACAA
	3# siRNA	GAAAUGGCAUUAUCCGAUU
ATG7	1# siRNA	GUGUCUAUGAUCCCUGUAA
	2# siRNA	CAUCAAUGCUGCGUUGGGA
	3# siRNA	CUACAAACUUGGCUGCUAU
P300	1# siRNA	GCACAAAUGUCCAGUUCUU
	2# siRNA	GCAUCAGAUCUGUGUCCUU
	3# siRNA	GCUACUGAAGAUCGAUUAA
Unrelated	Nontargeting Control siRNA	UUCUCCGAACGUGUCACGUTT

**Table 2:** Primers used in this study.

Primers	Sequence (5′→3′)	Length (nt)
LC3-F	ATGCCGTCCGAGAAAACCTTC	360
LC3-R	TCCGAAAGTCTCCTGAGAGGC	
ATG5-F	CAGCTCTTCCTTGGAACATCACA	241
ATG5-R	TCCATGAGTTTCCGATTGATGGC	
ATG7-F	CGGATGGTGAACCTCAGCGA	262
ATG7-R	CATACAGCGGCTGCCTCACAG	
β-actin-F	AGGCTCTCTTCCAACCTTCCTT	108
β-actin-R	CGTACAGGTCTTTACGGATGTCCA	
