# Supplementary material for: Autophagy Negatively Regulates Transmissible Gastroenteritis Virus Replication
Source: Sci Rep. 2016 Mar 31;6:23864. doi: 10.1038/srep23864 (PMC4814908; doi:10.1038/srep23864)
Supplement: Supplementary Information [file srep23864-s1.pdf]

Autophagy Negatively Regulates Transmissible Gastroenteritis Virus Replication

Longjun Guo[1, #], Haidong Yu[1, 2, #], Weihong Gu[1], Xiaolei Luo[1], Ren Li[1], Jian Zhang[1], Yunfei Xu[1], Lijun Yang[1], Nan Shen[1], Li Feng[1], Yue Wang[1, *]

[1] State Key Laboratory of Veterinary Biotechnology, Harbin Veterinary Research Institute, Chinese Academy of Agricultural Sciences, Harbin, China; [2] Weike Biotechnology, Harbin Veterinary Research Institute, Chinese Academy of Agricultural Sciences, Harbin, China

[#] These authors contributed equally to this work

[*] Address correspondence to Yue Wang, State Key Laboratory of Veterinary Biotechnology, Harbin Veterinary Research Institute, Chinese Academy of Agricultural Sciences, Harbin 150001; Email: wangyue@hvri.ac.cn

Fig. S1

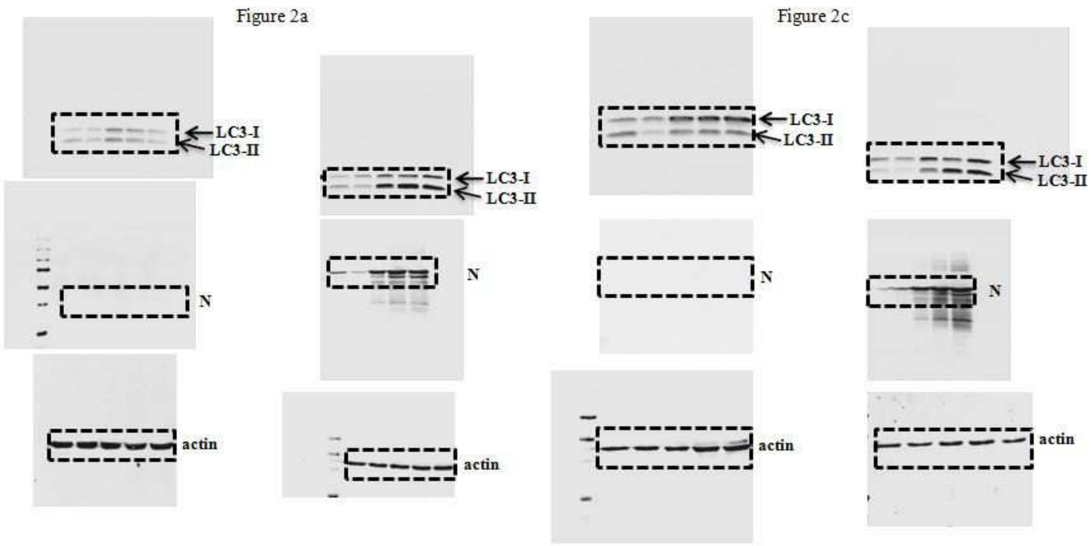

Fig. S1. Full-length images of the immunoblots in Fig. 2. Black dot line boxes indicate the cropped images used in Fig. 2.

Fig. S2

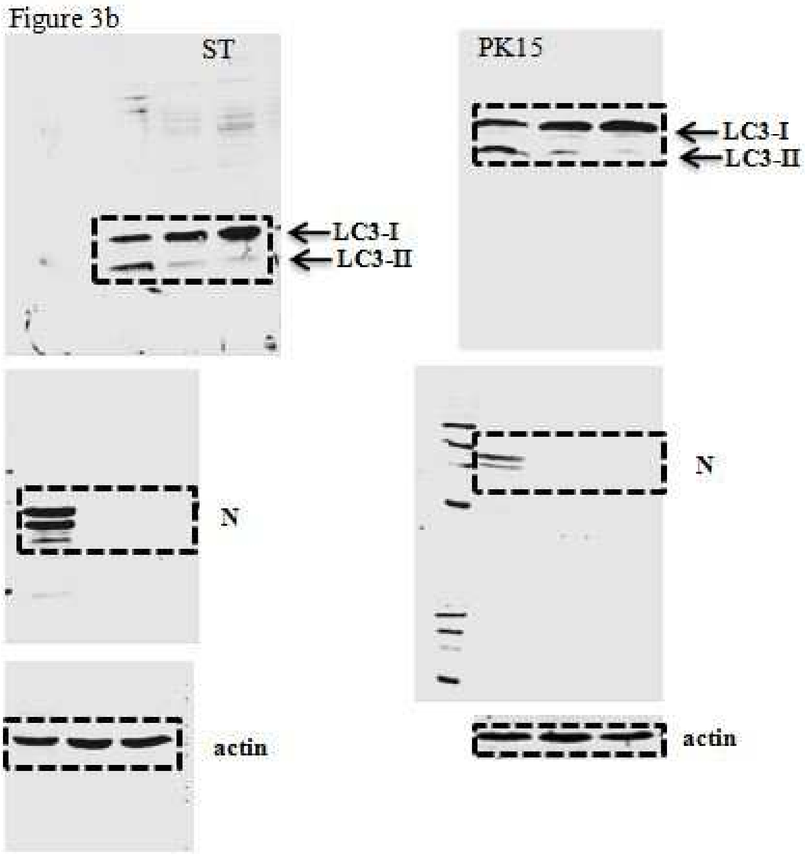

Fig. S2. Full-length images of the immunoblots in Fig. 3. Black dot line boxes indicate the cropped images used in Fig. 3.

Fig. S3

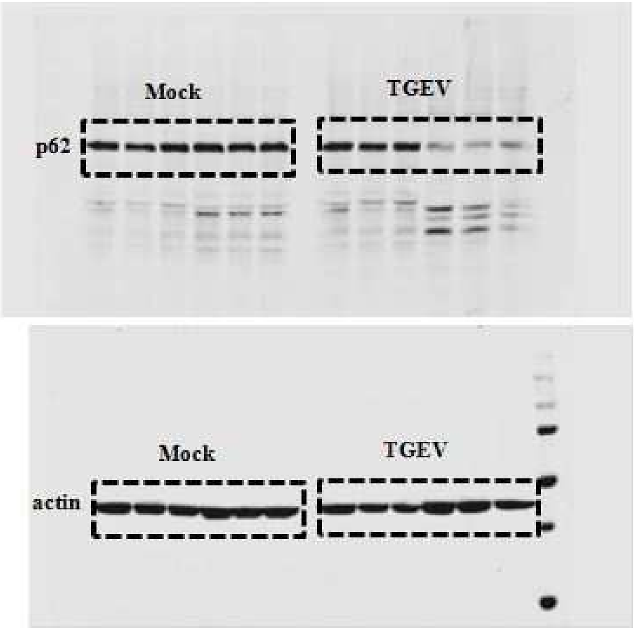

Fig. S3. Full-length images of the immunoblots in Fig. 4. Black dot line boxes indicate the cropped images used in Fig. 4.

Fig. S4

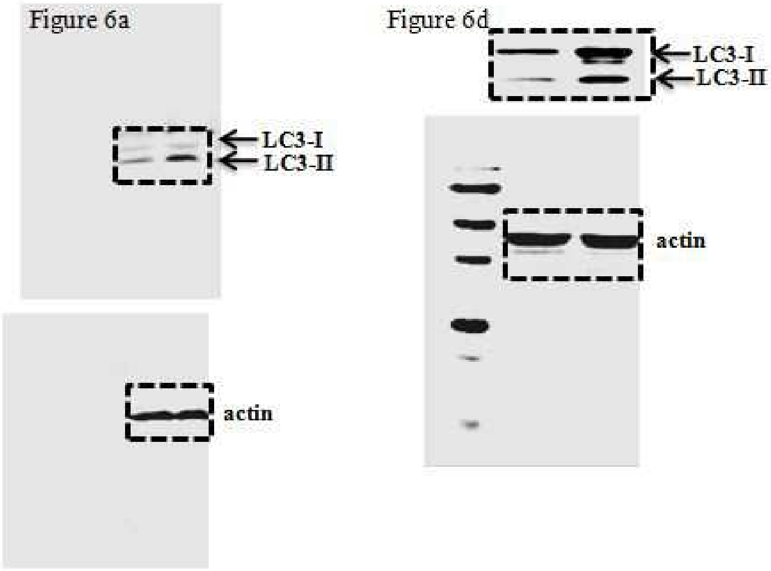

Fig. S4. Full-length images of the immunoblots in Fig. 6. Black dot line boxes indicate the cropped images used in Fig. 6.

Fig. S5

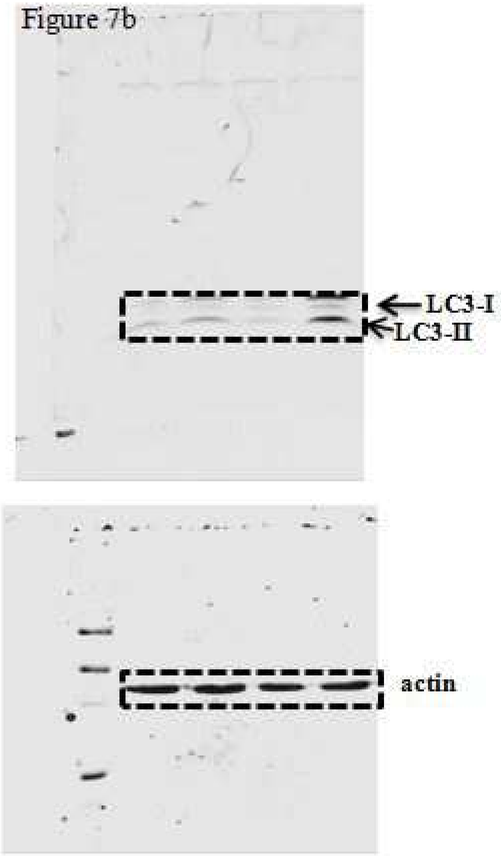

Fig. S5. Full-length images of the immunoblots in Fig. 7. Black dot line boxes indicate the cropped images used in Fig. 7.

Fig. S6

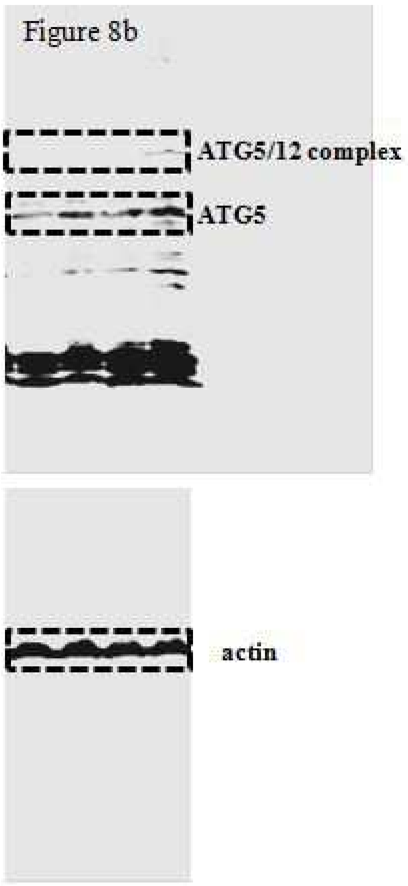

Fig. S6. Full-length images of the immunoblots in Fig. 8. Black dot line boxes indicate the cropped images used in Fig. 8.

Fig. S7

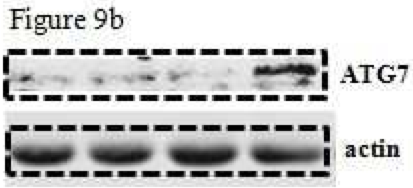

Fig. S7. Full-length images of the immunoblots in Fig. 9. Black dot line boxes indicate the cropped images used in Fig. 9.

Fig. S8

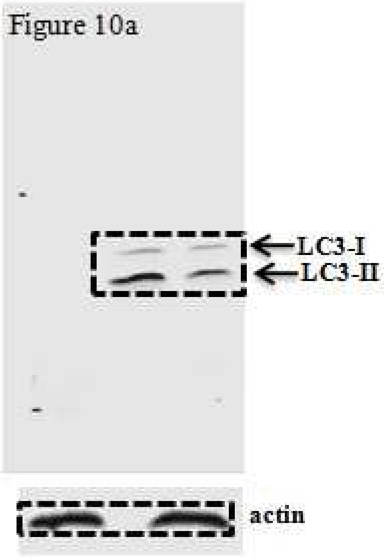

Fig. S8. Full-length images of the immunoblots in Fig. 10. Black dot line boxes indicate the cropped images used in Fig. 10.

Fig. S9

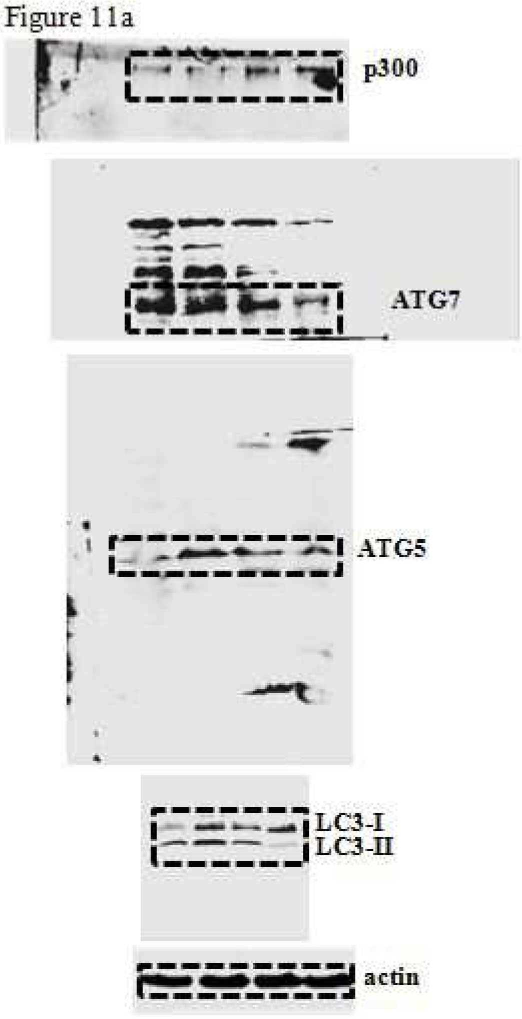

Fig. S9. Full-length images of the immunoblots in Fig. 11. Black dot line boxes indicate the cropped images used in Fig. 11.
